# Clinically Silent Amyloid-Related Imaging Abnormality With Edema Following Lecanemab Therapy: A Case Report

**DOI:** 10.7759/cureus.91230

**Published:** 2025-08-29

**Authors:** Imran Bitar, Mukhlis Alabdalrazzak, Mazen Zamzam, Yash Desai, Kamill Abushaban

**Affiliations:** 1 School of Medicine, Oakland University William Beaumont School of Medicine, Rochester, USA; 2 School of Medicine, Wayne State University School of Medicine, Detroit, USA; 3 Department of Radiology, Corewell Health William Beaumont University Hospital, Royal Oak, USA

**Keywords:** alzheimer's disease, amyloid-related imaging abnormalities, cortical edema, lecanemab, monoclonal antibody therapy

## Abstract

Amyloid-related imaging abnormalities with edema (ARIA-E) are a known complication of anti-amyloid monoclonal antibody therapies such as lecanemab. ARIA-E represents vasogenic cerebral edema resulting from treatment-related disruption of vascular amyloid and appears on MRI as cortical or gyriform T2 fluid-attenuated inversion recovery (FLAIR) hyperintensities. Clinically, ARIA-E ranges from asymptomatic radiologic findings to symptomatic events such as headache, confusion, or seizures, making routine surveillance important during therapy. We present the case of a 60-year-old woman with biomarker-confirmed AD who developed radiographically evident ARIA-E following six biweekly infusions of lecanemab. Surveillance MRI revealed new cortically based and gyriform T2 FLAIR hyperintensities in the posterior occipital and bilateral temporal lobes, consistent with parenchymal and sulcal edema. Notably, the patient remained neurologically asymptomatic throughout the episode. Lecanemab therapy was discontinued, and she was managed conservatively with close outpatient follow-up. This case highlights the importance of routine imaging during anti-amyloid therapy and demonstrates that conservative management may be appropriate in select asymptomatic cases of ARIA-E.

## Introduction

Alzheimer’s disease (AD) is a progressive neurodegenerative disorder characterized by cognitive and functional decline, with hallmark pathologic features including extracellular amyloid-beta (Aβ) plaque accumulation and intracellular tau neurofibrillary tangles [1]. In recent years, disease-modifying therapies targeting cerebral Aβ have become available, most notably lecanemab, an anti-Aβ42 immunoglobulin G1 (IgG1) monoclonal antibody approved by the U.S. FDA in 2023 for patients with mild cognitive impairment or mild dementia due to AD and confirmed cerebral amyloid pathology [2].

Lecanemab has demonstrated a significant reduction in amyloid burden and modest clinical benefit [3]; however, its use carries a recognized risk of amyloid-related imaging abnormalities with edema (ARIA-E), a form of vasogenic cerebral edema. ARIA-E typically occurs within the first several infusions, is more frequent in apolipoprotein E epsilon 4 (APOE ε4) carriers, and may range from asymptomatic radiologic changes to symptomatic neurologic events such as headache, confusion, or seizures [3-5]. MRI surveillance is therefore standard practice before and during anti-amyloid therapy [6].

We present a case of radiographically confirmed ARIA-E following six infusions of lecanemab in a 60-year-old woman with biomarker-supported AD. Despite radiologic findings consistent with parenchymal and sulcal edema in the posterior occipital and temporal lobes, the patient remained neurologically stable and was successfully managed with discontinuation of treatment and clinical monitoring. This case highlights the importance of routine MRI surveillance, the variable clinical expression of ARIA-E, and the role of conservative management in select presentations.

## Case presentation

A 60-year-old right-handed Caucasian woman with no significant medical history presented with a three-year history of insidiously progressive short-term memory decline, which began interfering with her occupational performance as a secondary-school teacher in 2022. Despite early retirement, she remained independent in all activities of daily living (ADLs), continued driving, and effectively managed medications and household finances using compensatory strategies developed during outpatient speech-language therapy.

Neurologic evaluation in March 2024 revealed mild temporal disorientation, impaired paragraph-level auditory comprehension, and reduced executive function. Her digit span was four forward and three backward. Speech was fluent with preserved repetition and naming, and both written language and object recognition remained intact.

**Figure 1:**
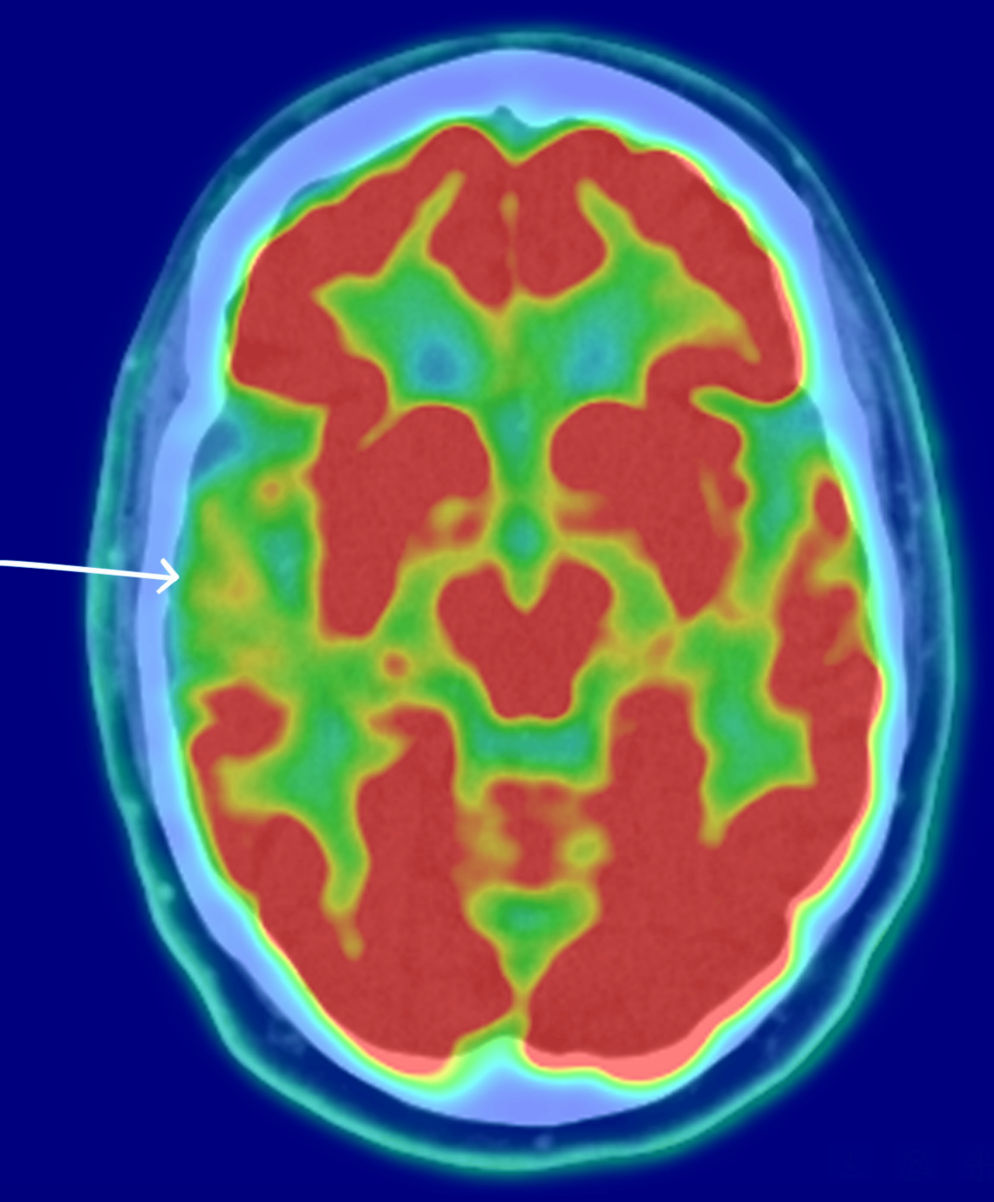
18F-FDG PET demonstrating right-predominant hypometabolism in the medial temporal and posterior parietal cortices with involvement of the cingulate and precuneus (white arrow). 18F-FDG PET: fluorine-18 fluorodeoxyglucose positron emission tomography

**Figure 2:**
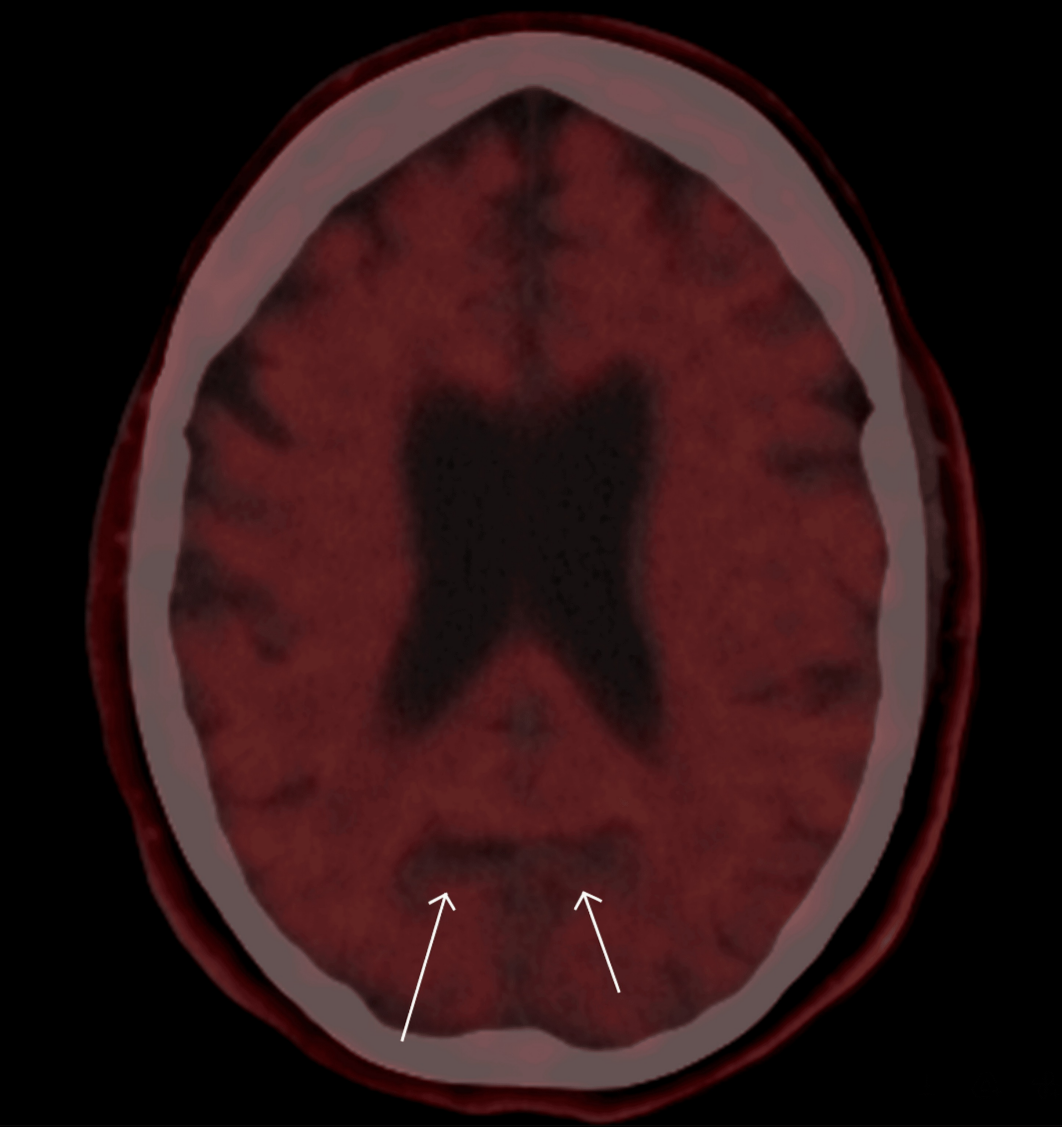
18F-florbetapir amyloid PET demonstrating diffuse cortical amyloid deposition, most evident as loss of gray-white matter contrast in the posterior and occipital lobes (white arrows).

A diagnostic biomarker work-up supported a diagnosis on the AD continuum. Fluorine-18 fluorodeoxyglucose positron emission tomography (18F-FDG PET) performed on March 11, 2024, demonstrated right-predominant hypometabolism in the medial temporal and posterior parietal cortices with involvement of the cingulate and precuneus, patterns classically associated with AD (Figure 1). A confirmatory 18F-florbetapir amyloid PET on July 16, 2024, showed diffuse cortical amyloid deposition, most evident as loss of gray-white matter contrast in the posterior and occipital lobes (Figure 2). Routine laboratory testing and structural MRI excluded reversible or alternative causes of cognitive impairment.

**Figure 3:**
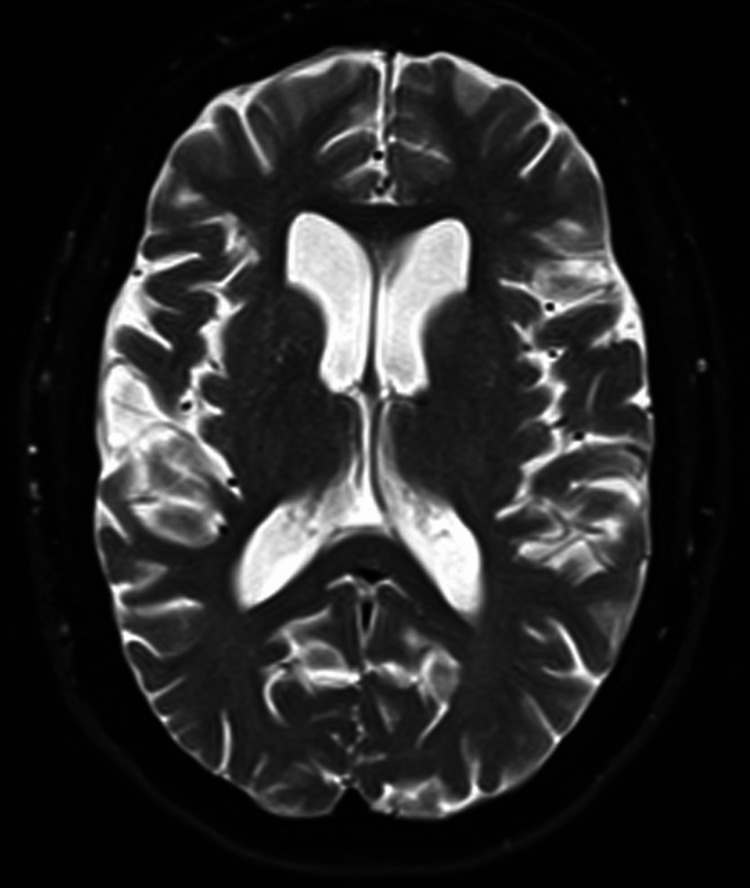
MRI showing generalized age-appropriate brain atrophy as evidenced by sulcal widening throughout the brain.

**Figure 4:**
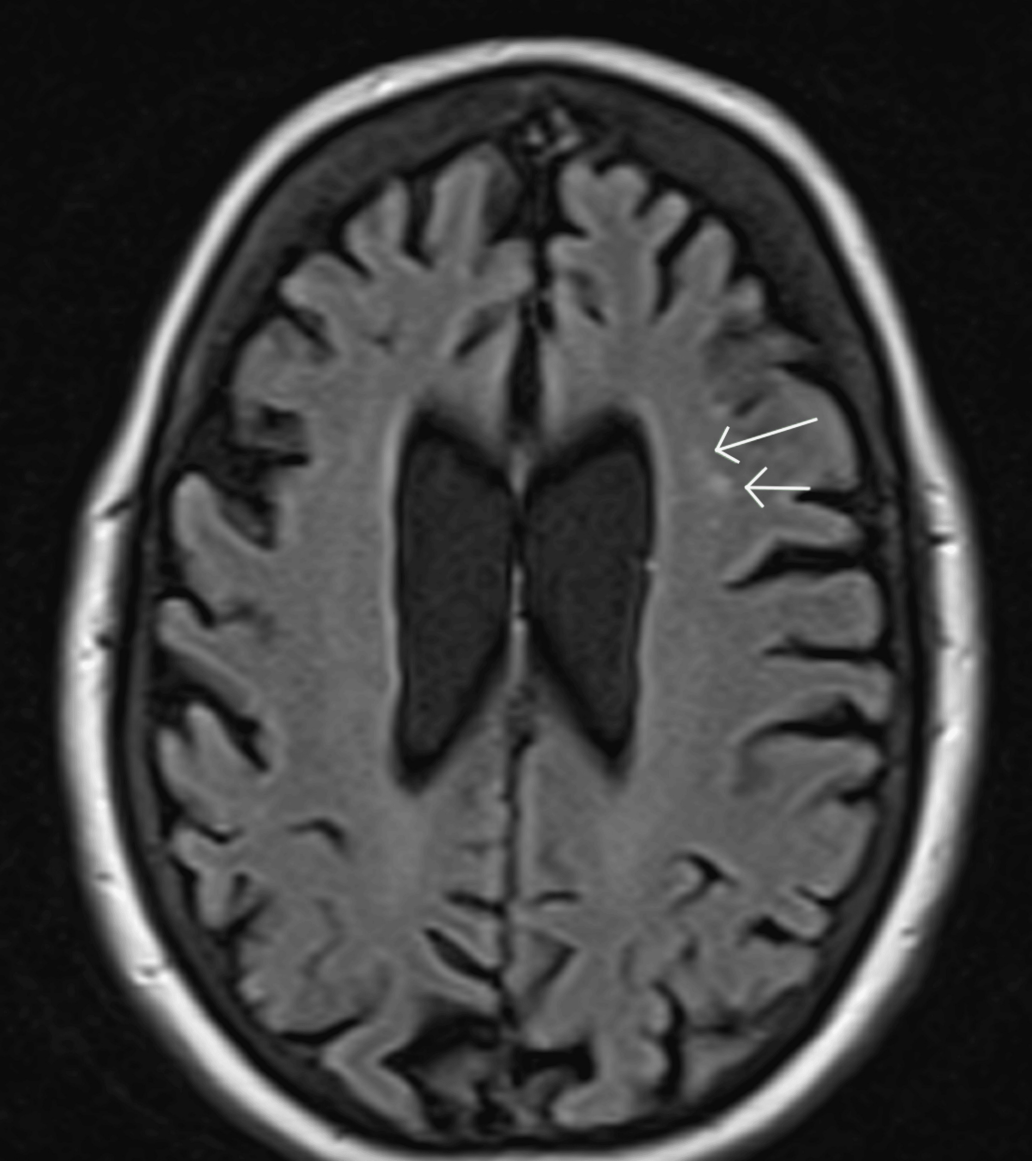
MRI showing small nonspecific T2-weighted hyperintensity in the left frontal lobe (white arrows).

In April 2025, the patient elected to initiate disease-modifying therapy with lecanemab, a U.S. FDA-approved anti-Aβ42 IgG1 monoclonal antibody for early AD. Treatment was administered at standard dosing via intravenous infusion every two weeks. She completed six infusions over three months. Given the known risk of ARIA-E, particularly within the first five infusions, baseline surveillance MRI was obtained on April 5, 2025, showing only age-appropriate atrophy (Figure 3) and a small nonspecific T2-weighted hyperintensity in the left frontal lobe (Figure 4).

**Figure 5:**
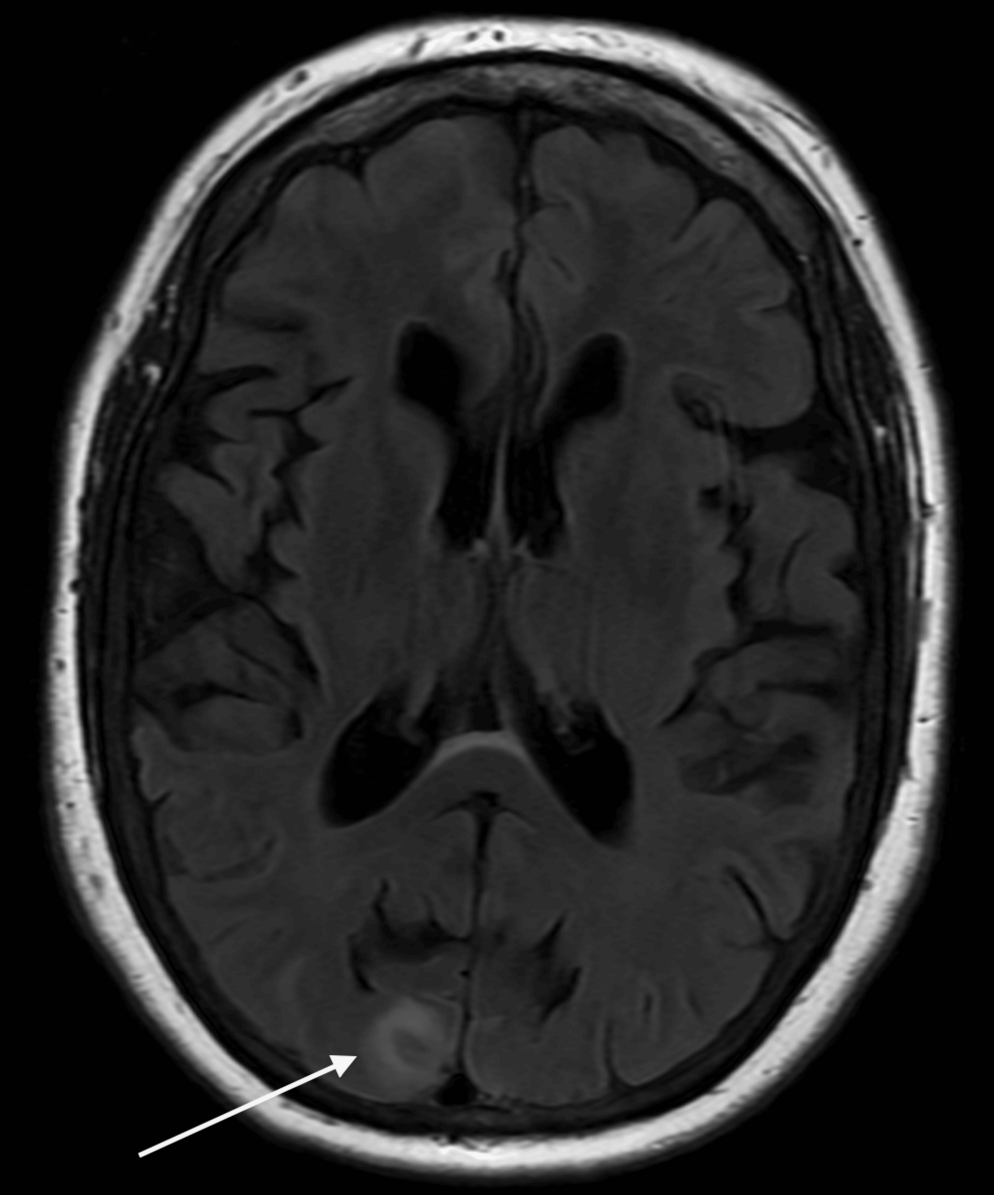
Axial T2 FLAIR MRI demonstrates a new cortically based hyperintense lesion in the right posterior occipital lobe (white arrow). This finding is consistent with parenchymal edema associated with ARIA-E, a known complication of anti-Aβ42 monoclonal antibody therapy. ARIA-E: amyloid-related imaging abnormalities with edema; FLAIR: fluid-attenuated inversion recovery

**Figure 6:**
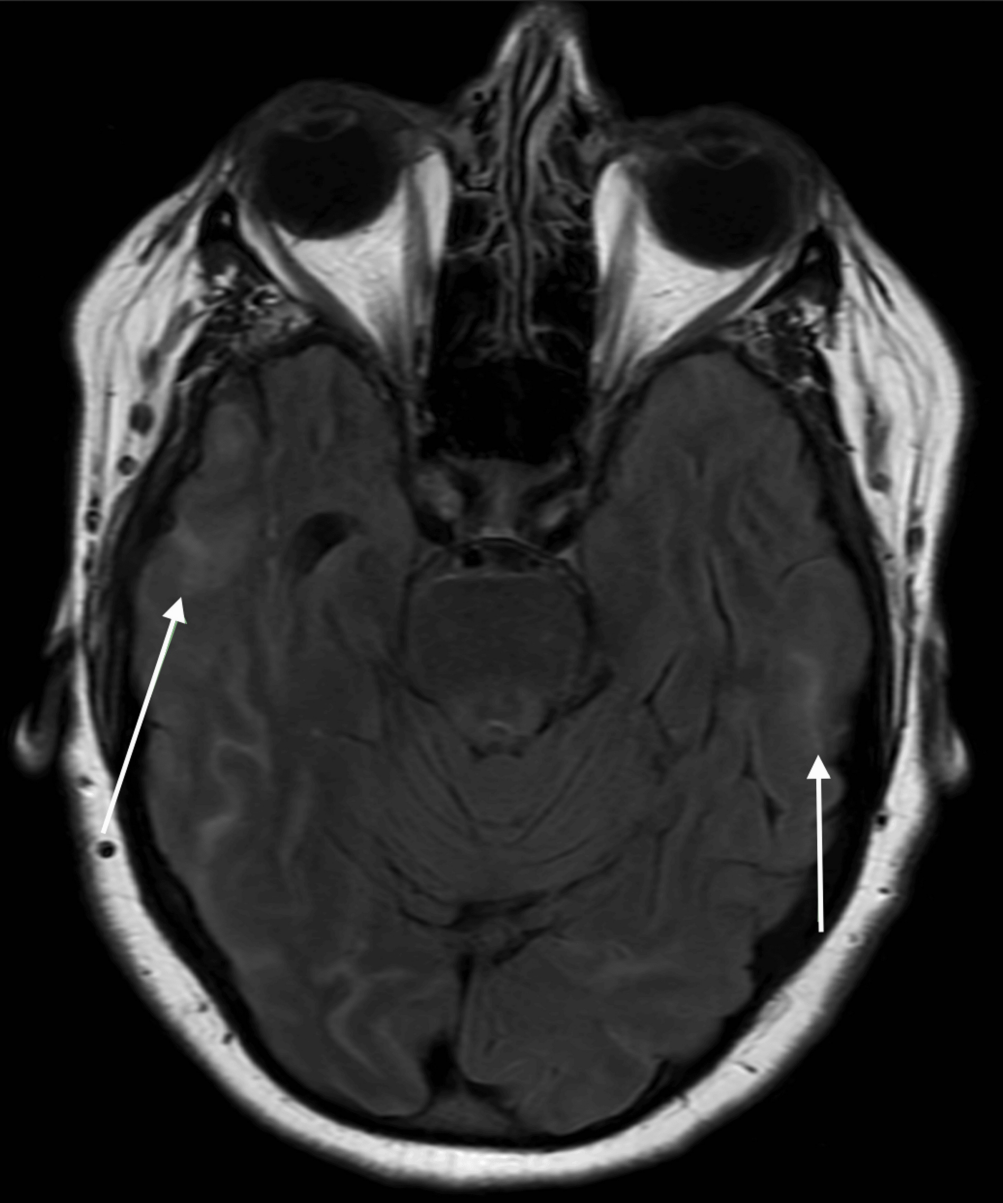
Axial T2 FLAIR MRI at the level of the temporal lobes shows bilateral curvilinear sulcal FLAIR hyperintensities (white arrows), more pronounced on the right. These gyriform signal changes are suggestive of sulcal edema, also consistent with ARIA-E in the setting of anti-amyloid therapy. No associated hemorrhage was observed on susceptibility-weighted imaging. ARIA-E: amyloid-related imaging abnormalities with edema; FLAIR: fluid-attenuated inversion recovery

Following the sixth infusion, a surveillance non-contrast MRI of the brain revealed new radiologic abnormalities: a cortically based T2 fluid-attenuated inversion recovery (FLAIR) hyperintense lesion in the right posterior occipital lobe (Figure 5) and additional curvilinear, gyriform FLAIR hyperintensities in the bilateral posterior occipital and temporal lobes, more prominent on the right (Figure 6). Susceptibility-weighted imaging (SWI) showed no evidence of intracranial hemorrhage. These findings were interpreted as cerebral edema consistent with ARIA-E.

Clinically, the patient remained stable without new neurologic deficits. She was managed conservatively with discontinuation of lecanemab and close monitoring. 

## Discussion

ARIA-E is a known complication of anti-amyloid monoclonal antibody therapies such as lecanemab and represents one of the principal safety considerations limiting broader therapeutic use. This case contributes to the growing clinical experience of ARIA-E, providing a valuable illustration of a radiographically confirmed but clinically asymptomatic episode following six infusions of lecanemab. The patient’s favorable outcome following drug discontinuation and conservative management is consistent with emerging reports suggesting that many cases of ARIA-E are self-limited and manageable without corticosteroids or hospitalization [7,8].

The incidence and severity of ARIA-E have been systematically studied in clinical trials. In the phase 2 study of lecanemab, ARIA-E occurred in 9.9% of treated patients, with the majority of events arising within the first three months, typically after the initial few infusions [7]. A comprehensive exposure-response analysis further confirmed this early onset pattern and demonstrated a dose-dependent relationship, with higher plasma exposure correlating with increased ARIA-E risk [9]. Notably, our patient’s radiographic findings emerged in the expected timeframe, a pattern frequently reported in the literature [7].

Genetic predisposition also plays a significant role in ARIA-E risk. Carriers of the APOE ε4 allele, especially homozygotes, experience a markedly increased risk of developing ARIA-E during treatment with anti-amyloid antibodies [10]. APOE ε4 has been implicated in weakening blood-brain barrier integrity, increasing vascular permeability, and exacerbating amyloid deposition in cerebral vessels, all of which predispose to ARIA development [11,12]. In this case, the patient was not a carrier of the APOE ε4 allele. This underscores that ARIA-E can occur in patients without the APOE ε4 allele, supporting the need for MRI surveillance in individuals undergoing anti-amyloid therapy.

Imaging surveillance is critical in the early detection of ARIA. T2-weighted FLAIR imaging remains the modality of choice, revealing characteristic gyriform or curvilinear cortical edema, frequently located in posterior cortical regions such as the occipital or temporal lobes [13]. The presented case aligns with this pattern, highlighting typical radiologic findings with clinical stability.

Management of ARIA-E depends on its severity and symptomatology. In asymptomatic patients with mild imaging findings, temporary suspension of therapy and observation is often sufficient. Multiple reports and reviews indicate that ARIA-E may resolve spontaneously within weeks to months without the need for corticosteroids or hospitalization [14,15]. In cases with more severe imaging abnormalities or clinical symptoms (e.g., headache, confusion, seizures), treatment discontinuation and corticosteroid therapy may be required [16].

## Conclusions

This case illustrates that ARIA-E can occur early during lecanemab therapy and may present without clinical symptoms despite significant radiologic findings. Routine MRI surveillance is essential for early detection, and in asymptomatic patients, conservative management with treatment discontinuation and monitoring may be sufficient. As anti-amyloid therapies become more widely used, recognizing the variable presentations and appropriate management strategies for ARIA-E will be critical for optimizing patient safety.
